# How feasible is it to abandon statistical significance? A reflection based on a short survey

**DOI:** 10.1186/s12874-020-01030-x

**Published:** 2020-06-03

**Authors:** Fredi Alexander Diaz-Quijano, Fernando Morelli Calixto, José Mário Nunes da Silva

**Affiliations:** 1 Department of Epidemiology, School of Public Health, University of São Paulo, Av. Dr. Arnaldo, 715, Cerqueira César, CEP 01246-904, São Paulo, SP 01246-904 Brazil; 2 Laboratório de Inferência Causal em Epidemiologia da Universidade de São Paulo (LINCE-USP), São Paulo, Brazil; 3 Public Health, School of Public Health, University of São Paulo, São Paulo, Brazil; 4 Epidemiology, School of Public Health, University of São Paulo, São Paulo, Brazil

**Keywords:** *p* values, Null-hypothesis, Statistical inference, Statistical significance

## Abstract

**Background:**

There is a growing trend in using the “statistically significant” term in the scientific literature. However, harsh criticism of this concept motivated the recommendation to withdraw its use of scientific publications. We aimed to validate the support and the feasibility of adherence to this recommendation, among researchers having declared in favor of removing the statistical significance.

**Methods:**

We surveyed signatories of an article published that defended this recommendation, to validate their opinion and ask them about how likely they will retire the concept of statistical significance.

**Results:**

We obtained 151 responses which confirmed the support for the mentioned publication in aspects such as the adequate interpretation of the *p*-value, the degree of agreement, and the motivations to sign it. However, there was a wide distribution of answers about how likely are they to use the concept of “statistical significance” in future publications. About 42% declared being neutral, or that would likely use it again. We described arguments referred by several signatories and discussed aspects to be considered in the interpretation of research results.

**Conclusions:**

The responses obtained from a proportion of signatories validated their declared position against the use of statistical significance. However, even in this group, the full application of this recommendation does not seem feasible. The arguments related to the inappropriate use of statistical tests should promote more education among researchers and users of scientific evidence.

## Background

The culture of testing the null hypothesis through the *p*-value has dominated the practice of statistical inference [1]. In this sense, the level of significance is defined according to the alpha error that we would be willing to accept when rejecting the hypothesis that there is no association between variables of interest [2]. This level (often set at 0.05) is used to define whether an association is “statistically significant” according to the *p*-value obtained from the tests [3].

However, the p-value varies depending on the sample size and the magnitude of the association, and the latter varies randomly even in the absence of biases. Due to a generalized application of the same level of significance, investigations on a particular subject can lead to different conclusions, especially with limited sample sizes or in the case of weaker associations [4–6]. Using lower levels of significance (e. g., 0.005 instead of 0.05) or calculating the posttest probability have been suggested to address lack of replication of the claimed associations [6–9].

With a more radical approach, an article recently published in the journal *Nature* called on the entire scientific community to abandon the concept of “statistical significance” in scientific publications. More than 800 signatories supported this paper [10]. Even though we agree with most of the arguments cited in this publication, we considered that the recommendation of entirely abandoning the statistical significance might be less useful than promoting its rational use.

**Fig. 1:**
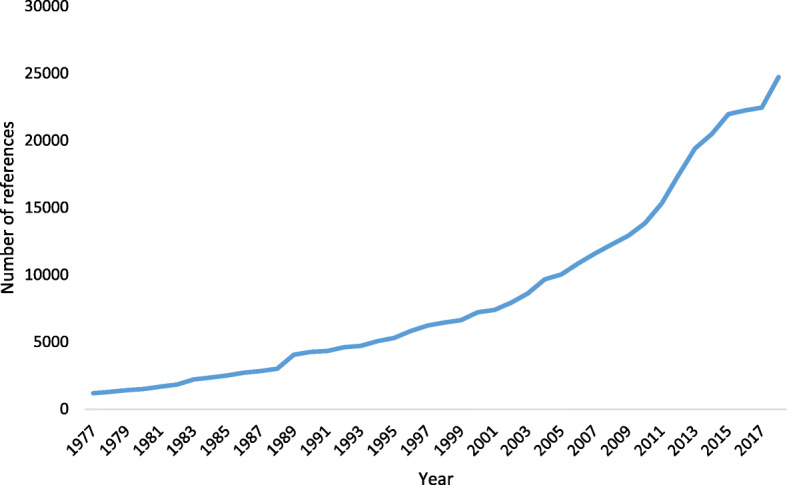
Count of references obtained in Pubmed with the term “statistically significant” (1977–2018)

Besides, there is a growing trend of using the term “statistically significant” in the biomedical literature, as seen in Pubmed searches (Fig. 1). Therefore, we wonder how feasible it would be to abolish the use of this concept from our future publications. For this reason, we conducted a short survey with the signatories of the article to remove the statistical significance to consult the probability of them not using this term anymore. Also, we considered pertinent to validate the support of these researchers for the recommendation mentioned above.

## Methods

We sent a short survey to each of the 854 signatories by email between May 5 and 10, 2019 (approximately six weeks after publication). The questionnaire (attached in the supplementary information) included three questions to avoid duplicate answers (country of residence, gender, and date of birth). Moreover, we added three questions to validate the support of the signatories:

- One of them presented a scenario for the interpretation of a value of p. This multichoice question had as a correct option one considering *p*-value as the probability of finding values at least as extreme as those observed, assuming that the null hypothesis was true. We also considered as right when participants did not check that answer but referred to a proper interpretation of the question in the comments.
- Another question aimed to confirm the support to the recommendation, which was formulated as “Currently, how much do you agree with the retiring of the statistical significance of future scientific publications?” The options were presented on a Likert scale with five possible responses.
- Also, we asked about factors influencing the decision to sign the paper on retiring of statistical significance. This question allowed choosing multiple answers from four suggested options (arguments against statistical significance, arguments in favor of alternative terms, the prestige of the authors and the prestige of the journals of the publication) and writing down other motivations.

In addition to these questions, we asked the signatories how likely they are to use the concept of “statistical significance” in their future publications. The options included:

- Never (I expect never to use it again)
- Unlikely (It is unlikely that I will use it again)
- Neutral, or it depends on the occasion
- Likely (It is likely that I will use it again)
- Always (I will use it every time I have the chance)

We presented the distribution of answers to this question as raw frequencies, and, to address potential biases from non-responders, we also calculated values weighted by the inverse the probability of responding. To calculate this probability by sex and country, we grouped the geographical origin in five categories for women and ten for men, including respectively the three and the eight countries with the highest number of responders and two regions grouping the others. Four participants (two men and two women) did not provide data from residence, so for them, we considered the average probability of response according to the sex category. We discounted the weight of these participants from the total and distributed the rest among the other participants into the corresponding sex category. In this way, the sum of all the weights remained equal to the number of signatories.

In the end, the questionnaire had an open question to record additional comments, and those we considered related to the discussion are presented in the supplementary material. We excluded any data that could potentially identify the respondent or another person. Since the questionnaire was anonymous, we were not able to send personalized reminders to the nonrespondent.

This study was evaluated and approved by the Ethics Committee of the School of Public Health of the University of São Paulo. A link for an informed consent form was sent to the participants in the invitation email. The survey was intended to be responded anonymously, and any data suggesting an individual identity was treated confidentially.

## Results

Out of the forms sent to all the 854 signatories, we received 153 responses. We excluded two (one because it claimed to be lying and another because it was considered duplicate), obtaining a total of 151 valid responses, mostly from men (*n* = 136) with a median age of 43 years old (interquartile interval: 36 to 56). By considering the characteristics of the total of signatories, we observed a higher response rate of men compared to women, and notable differences between countries (Table 1).

**Table 1:** Response rates according sex and country categories of signatories

Category	Signatories	Responders (%)
**Sex**		
Male	652	136 (20.8)
Female	202	15 (7.4)
**Country**		
USA	293	61 (20.8)
UK	84	9 (10.7)
Germany	54	12 (22.2)
Canada	49	11 (22.4)
Australia	45	8 (17.8)
Switzerland	37	5 (13.5)
Italy	18	6 (33.3)
Brazil	7	5 (71.4)
Other countries from Europe	148	18 (12.2)
Other countries	119	12 (10.1)
Unknow		4
**Total**	854	151 (17.7)

The *p*-value for the comparison of the response rates by sex was 0.0001 (Fisher’s exact test); and by countries was 0.0002 (chi-square)

About the question of interpretation of the value of p, we considered 136 correct answers (including 133 closed selections and three open responses), corresponding to 90.1% of the total of respondents. Five participants did not answer this question or make a justification. Ten participants wrongly answered this question: all of them were male and proceeded from nine different countries.

Relating to the current degree of agreement with the decision to withdraw the statistical significance, 98 (65%) answered that they strongly agreed, 49 (32%) partially agreed, three neither agreed nor disagreed, and only one indicated a strong disagreement. The last one referred that did “not agree with the title of the essay” and “it is unfortunate that the press and colleagues (…) are focusing on the title”.

Concerning the motivations to sign, the majority (142/151, 94%) answered that it was because of the arguments against statistical significance, followed by the arguments in favor of the alternatives (91/151, 60.3%). Only a minority of the respondents recognized that the prestige of the authors (*n* = 9) or of the journal (*n* = 12) were part of the motivations; however, for none of them, this was the only motivation. Additionally, 20 respondents reported other motives such as problems of misinterpretation and misuse of *p*-values.

**Fig. 2:**
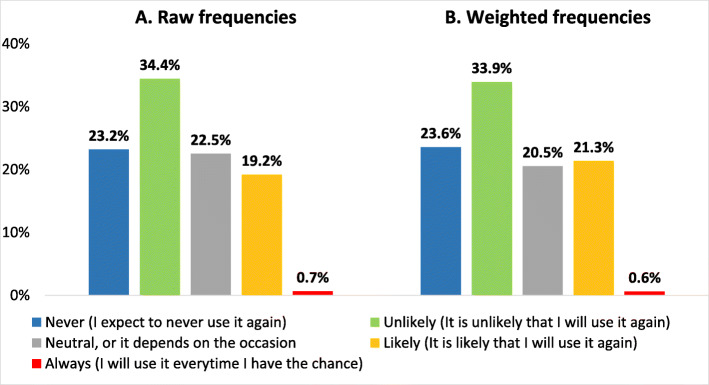
In your future publications, how likely are you to use the concept of “statistical significance”?

Regarding the probability of using the concept of statistical significance in future publications, 35 (23%) responded that they expect never to use it again; and, 52 (34%) said it would be unlikely. On the other hand, 34 (23%) answered as neutral, or it depends on the occasion; 29 (19%) indicated that it would be likely to be used again and one stated that they would use it whenever they had the opportunity (Fig. 2a). We obtained similar results when we weighted the frequencies by sex and the countries of residence (Fig. 2b).

We received several comments about the matter (supplementary material), of which we highlighted the following:

- "*"Significance" with firm thresholds is the problem. The credibility of a result is multi-determined. P-level is one of the determinants, but only one.*
  *The main - really the only - thing that’s needed to determine the credibility of a result is replication. There is no shortcut; you can’t know what the study would find if you repeated it, unless you repeat it. The use of "significance" and even exact p-levels typically is an attempt to avoid this stubborn truth*."
- "*Although I signed in agreement with the article, I do not think the title was properly reflecting the spirit with which it was written. We are not advocating to drop statistical significance altogether, but to make a more mindful use of it. The main mistakes are 1) to think the p-value gives us a measure of the strength or magnitude of a relationship, for example. 2) a p value can help use supporting or rejecting alternative hypothesis. We need to incorporate measures that make sense in the system we are studying. Effect sizes, confidence intervals, Bayesian or Information Theory approaches in addition to the classical stats*."
- "*It is impossible to interpret a p-value in the absence of some prior estimate of the probability of the null hypothesis being true (or false). I am much more in favor of presenting the Bayes Factor Bound*."
- "*The paper in question proposed to stop using the term "statistical significance". It did NOT propose that p values should be banned, but only that they should not be dichotomized. I proposed that p values should be supplemented by a number that represents the risk that a "positive" test is a false positive*."

## Discussion

This independent survey may be considered a validation of the support of a group of researchers to a recommendation to abandon the use of the concept of statistical significance. Most of the signatories correctly interpreted the *p*-value. This result is not superfluous because some studies suggest that the misinterpretation of the p-value can be frequent even among academics [11–13]. Besides, most responders strongly agree to abandon the use of “statistical significance” and, for the most part, were motivated by the arguments presented against this concept.

However, regarding the feasibility of abandoning statistical significance, close to a quarter are fully convinced that they will never use this concept again. On the other hand, about 42% declared being neutral or that would likely use it in future publications. Assuming that the researchers surveyed represent those against the concept, the distribution of answers to this question suggests that the fully retire of the statistical significance does not seem feasible. Although there were evident differences in the response rate according to the sex and country categories, the weighting for these variables led to similar results concerning this question. Therefore, we considered that this finding would not be explained by selection bias.

Because we were looking for a high response rate in our survey, we did not include questions related to the causes for which signatories would again use statistical significance in future publications. Therefore, the fact of using the concept of statistical significance does not mean that they are going to base their conclusions solely or primarily on this result. Also, it is possible those continuing to use this term would be motivated by compliance the expectations of journals, reviewers, or readers, more than by their way of interpreting the results.

The *p*-value will continue to be presented, and dichotomization results seem to be inevitable regardless of the criterion chosen. Despite this, based on the validation we have made, we consider that Amrhein et al. discerned in their paper a legitimate concern of researchers from different areas [10]. In that sense, we agree with the importance of a research finding not being based only on statistical significance [14, 15].

An aspect to highlight is to differentiate the application scenarios from statistical significance [12]. For example, there is a critical distinction between studies of causal inference vs. those for prediction purposes. In the latter, the interpretability of the estimates may be optional, and the statistical criteria can command decisions to use or not a predictor [16, 17].

However, in studies of causal inference, the concept of statistical significance should not be a primary concern. In those cases, the efforts must focus on adequately research designing and analysis to avoid bias, control confusion, and consider eventual effect modifications [18, 19]. After that, the measures of association and impact should define when a result is significant in the clinical and public health scopes [20].

Therefore, it is not surprising that one of the major concerns expressed by several of the signatories is the misuse and misinterpretation of the value of p. Also, well-documented publication biases in favor of “positive results” are a consequence of overvaluation of statistical significance [21, 22]. These are often concerns among editors and statistical consultants of biomedical journals. For example, *The New England Journal of Medicine* recently modified its guidelines for statistical reporting by including a requirement to replace *P* values with estimates and confidence intervals when neither the protocol nor the analysis plan has specified methods to adjust for multiplicity [23].

We agree that the value of a result must be based on the interpretation of the spectrum of values compatible with the data, such as Amrhein et al. suggested [10]. However, removing a term such as statistical significance is far from a solution to avoid the publication biases. We regret that, even based on point and interval estimates, associations compatible with the null value undoubtedly would continue being under-reported. Conversely, the absence of a preset threshold to interpret a *p*-value could increase subjectivity [24].

Faced with the seemingly inevitable use of statistical significance [21], we must give due value to statistical tests, promoting the understanding of their limitations [25]. In that sense, one critical issue is the widespread application of an arbitrary significance level (i.e., 0.05) [12, 26]. As an analogy, diagnostic tests may need different cut-offs according to the disease prevalence to maintain high predictive values [27]. Similarly, it would be negligent in using the same significance level for all research problems. The pre-test probability of an association would help to define a cut-off to increase the chance of both identifying the true associations and discarding those spurious [7]. Nevertheless, no value should become a new thumb rule applicable to all situations.

Reducing the significance level can reduce the false positive rate but increase the false negative rate, which is reducing the power of a study. This can be a problem when decisions have to be based on studies with small sample sizes, such as in preliminary outbreak investigations or in the research of extremely rare but severe diseases.

For another purpose, a study aimed to replicate or confirm results from other well-designed studies would not need to use the same level of significance, since the state of knowledge has changed. A higher significance level could be justified when previous studies suggested a high pre-test probability.

Moreover, other issues may be necessary to consider in each case, such as the implications of a false positive and false negative result. For example, it does not seem sensible to use the same significance level to approve a drug with a high risk of adverse effects as for a low-risk educational intervention. Probably in the former, we were more interested in ruling out the alpha error. As with diagnostic tests, the cut-off point for significance should also be adjusted to increase the likelihood that our research will cause more benefit than harm.

For all the above, it is likely that we have not yet found a magical formula to choose levels of significance. Therefore, we share the frustration of decisions being guided by an arbitrary or poorly justified rule. Statistical significance may play a supporting role, but not a leading one. However, better than trying to abolish this concept, we consider it is necessary to develop strategies to justify and predefine the significance levels considering both the evidence and the implications of errors resulting from the statistical tests. Moreover, efforts to define what is clinically or epidemiologically significant may be more useful to guide research and interventions [18, 19].

## Conclusions

The responses obtained from a proportion of signatories validated their declared position against the use of statistical significance. However, even in this group, the full application of this recommendation does not seem feasible (at least shortly). The arguments against the inappropriate use of statistical tests should promote more education among researchers and users of scientific evidence. Probably, the main problem does not rely on choosing a cutoff for the *p*-value, but on our difficulty recognizing the limitations of both statistics and rules.

## Supplementary information

**Additional file 1.** Questionnaire. Survey regarding the recommendation for retiring the statistical significance from scientific publications.

**Additional file 2.** Supplementary material. Comments related to the subject.

## Data Availability

All data generated and analyzed during this study are included in this published article and its supplementary material file.
