# Supplementary material for: How feasible is it to abandon statistical significance? A reflection based on a short survey
Source: BMC Med Res Methodol. 2020 Jun 3;20:140. doi: 10.1186/s12874-020-01030-x (PMC7271502; doi:10.1186/s12874-020-01030-x)
Supplement: Supplementary file 1 — Additional file 1. Questionnaire. Survey regarding the recommendation for retiring the statistical significance from scientific publications. [file 12874_2020_1030_MOESM1_ESM.pdf]

Survey regarding the recommendation for retiring the statistical significance from scientific publications

You have been invited to participate in this survey as a signatory of the paper "Scientists rise up against statistical significance" recently published in Nature (21 March 2019; Vol 567, p.305-307).
This survey was designed to be responded anonymously. However, you can send us any comment which will be treated confidentially.
* Required

1. Country of residence *

____

2. Gender *

Mark only one oval.

- ⬭ Female
- ⬭ Male

3. Date of birth

____

Example: January 7, 2019

4. Currently, how much do you agree with retiring of statistical significance of future scientific publications? *

Mark only one oval.

- Strongly agree with the retiring
- Partially agree
- Neither agree nor disagree
- Partially disagree
- Strongly disagree

5. In your future publications, how likely are you to use the concept of "statistical significance"? *

Mark only one oval.

- Never (I expect to never use it again)
- Unlikely (It is unlikely that I will use it again)
- Neutral, or it depends on the occasion
- Likely (It is likely that I will use it again)
- Always (I will use it everytime I have the chance)

6. Which of the following factors influenced your decision to sign the paper on retiring of statistical significance? (you can select more than one) *

Check all that apply.

- [ ] arguments against the use of statistical significance
- [ ] arguments in favor of the use of alternative concepts
- [ ] the prestige of the authors of the publication
- [ ] the prestige of the journal (Nature)

Other: [ ] ____________________

7. Assuming the absence of biases and confounding, consider a p-value of 0.06. Which of the following interpretations would be the most appropriate?

Mark only one oval.

- ( ) If the null hypothesis is true, the estimated probability of obtaining results at least as extreme as observed is 6%.
- ( ) If the alternative hypothesis is true, the estimated probability of obtaining results at least as extreme as observed is 94%.
- ( ) The estimated probability that the null hypothesis is true is 6%.
- ( ) It would be expected to replicate this result in 94% of the studies.
- ( ) If I reject the null hypothesis, there is a 6% probability that I am making a mistake.

8. Please feel at ease to send us any comment (optional).

This content is neither created nor endorsed by Google.

Google Forms
