# Supplementary material for: How feasible is it to abandon statistical significance? A reflection based on a short survey
Source: BMC Med Res Methodol. 2020 Jun 3;20:140. doi: 10.1186/s12874-020-01030-x (PMC7271502; doi:10.1186/s12874-020-01030-x)
Supplement: Supplementary file 2 — Additional file 2. Supplementary material. Comments related to the subject. [file 12874_2020_1030_MOESM2_ESM.docx]

**Supplementary material**

**Comments related to the subject.**

| “ *"Significance"* with firm thresholds is the problem. The credibility of a result is multi-determined. P-level is one of the determinants, but only one.  The main -- really the only -- thing that's needed to determine the credibility of a result is replication. There is no shortcut; you can't know what the study would find if you repeated it, unless you repeat it. The use of "significance" and even exact p-levels typically is an attempt to avoid this stubborn truth.” |
| --- |
| “Although I signed in agreement with the article, I do not think the title was properly reflecting the spirit with which it was written. We are not advocating to drop statistical significance altogether, but to make a more mindful use of it. The main mistakes are 1) to think the p-value gives us a measure of the strength or magnitude of a relationship, for example. 2) a p value can help use supporting or rejecting alternative hypothesis. We need to incorporate measures that make sense in the system we are studying. Effect sizes, confidence intervals, Bayesian or Information Theory approaches in addition to the classical stats.” |
| “…The article [Amrhein, V., et al. Retire statistical significance. *Nature* 2019; 567: 305–307] actually addresses the (mis)use of the label statistical significant to justify proving or disproving hypotheses, etc…” |
| “I am not opposed to p-value or significance testing. I am opposed how it is being used in social sciences. There are gross violations of the underlying assumptions. It seems to me that most of the researchers are using p-value blindly; they lack the mathematical skills.” |
| “… I am not opposed to reporting p-values. I am not opposed to using the language of statistical significance. I am in favor of no longer using statistical significance as a criterion in making publication decisions. Unfortunately, these issues have not been clearly separated.” |
| “I did not agree with the title of the essay and it is unfortunate that the press and colleagues (your survey included) are focusing on the title. No one with some experience in stats is in favor of "retiring" the concept, in fact the ASA recommendations speak of the need to make statistical significance one more element to consider. What must stop is that people are not thinking or consider the evidence first, only then making inferences from the data.” |
| “I do believe that, if conducted correctly (i.e. preregistration of hypothesis, analytic plan and sample size as per power analysis), the use of significance testing may be justified. This is why I answered that, depending on the occasion, I might still use it.” |
| “I don't think any are that appropriate - I would use the credibility (confidence) intervals and point estimate to appropriately interpret the results. If I were to say anything about the p-value - using above example - it would be the first one but that is not useful alone” |
| “I feel that adopting "results blind publishing" may be the most useful approach to solving some of the problems cited in this regard. I feel it is worth trying as an experiment. “ |
| “ … I've also been loosely following the misuse of statistics in psychology since 2000 via colleagues” ... “There's a cost to dropping a standard bar, but I agreed with the petition's authors that the binary significance distinction is now doing more harm than good. Arguably this is due to misuse - but after 70+ years it's clear that working scientists will continue to misuse them.  Will I abide by the recommendations? In the past I've tried a couple of times to avoid significance claims, or introduce more informative nulls. But there's been strong editor pressure to conform to editor/reader expectations, and in the end I wanted to get the paper published. But with more options available now, and pre-registration to remove the suspicious I picked the test to get the answer, and having now signed the petition, I hope at least to avoid dichotomous claims, and better emphasize effect sizes and reproduceability.”   “… we should focus on finding a reliable process that limits error rates, whatever our statistical persuasion. Instead of tossing my intriguing N=30 result into the void, it would be far better to wait until I could demonstrate a repeatable process with sustained low error rate. In that world, 5% might be a fine bar…” |
| “I think the survey questions are missing the main point that the letter had for me: statistical significance itself can be fine in specific settings, but the routine focus on dichotomous decisions (and calling those statistically significant) in publications is harmful to scientific communication.” |
| “I would favour plotting the frequentist posterior probability distribution of replication and marking on it P values, CIs etc. See: https://journals.plos.org/plosone/article?id=10.1371/journal.pone.0212302” |
| [other researcher] “… already tried to double-check the co-signatories; authors and co-signatories were nonplussed. Of course someone will "fail" one or more questions; testing a null hypothesis has no use here. You have got to approach this as an estimation problem; but do you happen to have a pre-determined cutoff for how many are too many? Even if nobody knew what they were signing, does that mean that the argument is wrong? I guess you are not running a similar survey with supporters of null hypothesis significance testing...” |
| “It is impossible to interpret a p-value in the absence of some prior estimate of the probability of the null hypothesis being true (or false). I am much more in favor of presenting the Bayes Factor Bound.” |
| “It might seem weird that I strongly agree with retiring statistical significance from future publication yet I will likely use it again. The reason is sadly simple: I work in a team, and when your colleagues don't necessarily share your view, you fall back to the most common practice, even though it is poor practice. This is especially true hen working with medical doctors, who are not statisticians and who consider this debate is statistical nonsense.” |
| “My answer to the last question shall be the most appropriate according to statistical methods” [regarding question about interpretation of p-value]. “Yet in the praxis, even the last answer (although less appropriate) would hold, despite being wrong from the methods (philosophical) point of view.” |
| “P-values are so much a part of current scientific communication that I am not hopeful that they will fade away anytime soon. I do believe that there are more logical and consistent statistics for measuring strength of evidence. My personal preference is to use second generation p-values (Blume et al. Am Stat. 2019;73(sup1):157-67). However, likelihood ratios or Bayes factors are also a reasonable alternatives.” |
| “… I am a statistical modeller and I believe all the uncertainty should be considered in a data analysis. Conclusions regarding the study should not be based on statistical significance, the statistical inference process should be completely reported (in supplementary material if necessary) and decisions should be made not only with statistical results but also with some scientific support (support on the literature, similar studies, systematic reviews and meta analyses, and so on). Sometimes a p-value of 0.06 is enough to suggest an association, and sometimes a p-value of 0.0001 is not enough (the existence of Boson Higgs in physics for instance they need a p-value < 0.0000003 to say there is some evidence of existence of Boson Higgs)…” |
| “The main reason I signed is that significance thresholds currently causes a bias of published research by specification searches trying to achieve certain significance levels” |
| “The paper in question proposed to stop using the term "statistical significance". It did NOT propose that p values should be banned, but only that they should not be dichotomized. I proposed that p values should be supplemented by a number that represents the risk that a "positive" test is a false positive.” |
| “The thrust of the Comment in Nature, and my own opinion, is not to fully abandon significance testing: rather it is to take a more nuanced approach where we don't set an arbitrary threshold p-value as a yes/no on "truth" as is unfortunately often seen. p values, magnitudes, and uncertainties are all important...” |
| “The very point of the statement is the "absence of bias and confounding" is a state that nearly never occurs in scientific reality, and that any number of violated assumptions even in cases of RCTs, will invalidate that interpretation or be tenable only for a weak hypothesis (e.g. ITT / average causal effect of randomization). In most settings, it is much more valuable to focus on the peculiarities of estimation, including issues of targets for inference, selection bias, measurement error, improper modeling, and sensitivity analyses. By focusing on a singular threshold for interesting results, and thereafter publication, I have seen first-hand how it cripples research projects by encouraging step-wise variable selection, hypothesis free stratification/interaction testing, overlooking extreme qualitative interactions because of borderline p-values, emphasizing apparent interactions that arise due solely to the lack of data support or poor modeling choices, and so on. While testing retains usefulness in experiments and replication, it is by and large a crutch that supports poor empirical research practices.” |
| “... The problems are well-known and it was a good opportunity to raise the issue. Even people who do not have expertise or do not know what the p-value is, have heard of the problem and want a solution. p-values are still informative and I am also ok with making comments about statistical significance, I said it is likely to use the term, though I would prefer not because I know my collaborators and journals will insist. what I am not ok with is when whole papers boil down to a p-value and people judge the p-value and whether is below or above 5% and not the actual effect size” |
| “Whether I use statistical significance indications depends strongly on Journals' conventions. If they ask me for "little stars" I will provide them but not put emphasis on their interpretation. Generally I believe that the problem goes much deeper, at least in my field of economics and organization studies. It is generally not sufficiently understood that we need to KNOW data generation processes (models) in order to identify or test individual parameters, while if we know parameter values as they SHOULD be, we test the model as a whole. It has to be understood that we cannot do both simultaneously ...” |
| “Why condition your results on the notion that the null hypothesis is true, and calculate a probability for obtaining data that you DID obtain? Why not assume the data you obtained are true (you did obtain them, after all, so their existence has a Pr = 1!), and quantify the probability of various hypothesis? Bayes Theorem is your friend!” |
